# Supplementary material for: Highly Sensitive ZnO/Au Nanosquare Arrays Electrode for Glucose Biosensing by Electrochemical and Optical Detection
Source: Molecules. 2023 Jan 7;28(2):617. doi: 10.3390/molecules28020617 (PMC9861633; doi:10.3390/molecules28020617)
Supplement: Supplementary file 1 [file molecules-28-00617-s001.zip › molecules-2115048-supplementary.pdf]

Supplementary Materials (SM)

Highly Sensitive ZnO/Au Nanosquare Array Electrode for Glucose Biosensing by Electrochemical and Optical Detection

Vinda Zakiyatuz Zulfa [1], Nasori Nasori [1,*], Ulya Farahdina [1], Miftakhul Firdhaus [1], Ihwanul Aziz [2], Hari Suprihatin [2], Muslikha Nourma Rhomadhoni [3] and Agus Rubiyanto [1]

[1] Laboratory Medical Physics and Biophysics, Department of Physics, Faculty of Sciences and Data Analytic, Sepuluh Nopember Technology Institute, Surabaya 60111, Indonesia

[2] Research Center for Accelerator Technology, Research Organization of Nuclear Energy, National Research and Innovation Agency (BRIN) Yogyakarta 55281, Indonesia

[3] Occupational and Safety Department, Nahdlatul Ulama University of Surabaya, Surabaya 60237, Indonesia

* Correspondence: nat.nasori@physics.its.ac.id

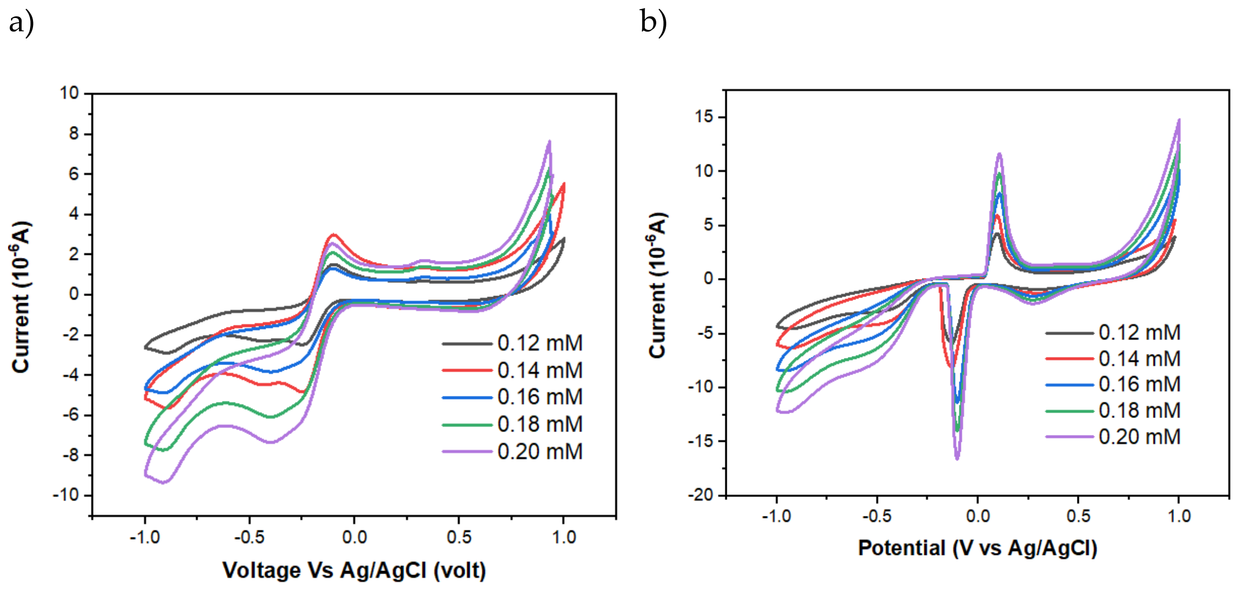

Figure S1. CV graph of the ZnO/Au nanosquare array with the addition of GOx with varying concentrations of a) fructose and and b) sucrose.
